# Doing public health differently: How can public health departments engage with local communities through social media interventions?

**DOI:** 10.1016/j.puhip.2023.100412

**Published:** 2023-07-21

**Authors:** Megan Watkins, Jaimee S. Mallion, Daniel Frings, Jane Wills, Susie Sykes, Andrew Whittaker

**Affiliations:** a Institute of Health and Social Care, London South Bank University, United Kingdom; b School of Applied Sciences, London South Bank University, United Kingdom

**Keywords:** Online community, Facebook, Public health, COVID-19, Collaboration

## Abstract

**Objectives:**

This paper evaluates a collaborative intervention between public health professionals and local social media administrators, in which the social media site Facebook was used with a view to strengthening engagement with and, dissemination of, core messages and building trust and resilience within local communities during the COVID-19 pandemic.

**Study design:**

A qualitative design was used, exploring the research question: how does collaboration between public health professionals and local social network group administrators create community engagement during a global crisis?

**Methods:**

Fourteen semi-structured interviews were conducted with public health staff and online group administrators. Data was analysed using framework analysis.

**Results:**

Collaboration between public health professionals and local group administrators created both opportunities and challenges. Local group administrators had wide reach and trust within the local community, but message credibility was enhanced through local authority involvement. Such collaborations contain inherent tensions due to perceived risks to social capital and independence but can be successful if receiving strong risk-tolerant support from the local authority. Findings are discussed in the context of Bourdieu's theory of social capital to examine how public health information can be delivered by trusted social media actors in communication tailored to the local community.

**Conclusions:**

Social media provides new channels of communication for delivery of public health messages, enabling new ways of working which create long-term engagement and community building. Although the intervention was developed quickly in response to the COVID-19 pandemic, participants felt it could be mobilised to address a wider range of issues.

## 1 Introduction

The COVID-19 pandemic presented considerable challenges to public health professionals engaging with communities. At a time where reliable information was needed, public health professionals found themselves cut off from face-to-face contact with communities, whilst community members were also isolated from one another. During the pandemic, community engagement became especially important to reach marginalised communities [1]. There is growing recognition that social media offers opportunities to engage with the public in new ways and that public health organizations need to learn to be more “social” [2]. While social media is often identified as a threat due to the dissemination of misinformation [3], there is evidence that social media provides a positive means of promoting health literacy [4]. It can be argued that social media allows for anonymised participation and equitable access. For example, a systematic review found social media interventions reduce health inequalities for younger people, older people, people in rural settings and with low socioeconomic status [5].

The potential for social media to act as a facilitator or barrier for public health efforts has been recognised in the ‘SPHERE’ framework which illustrates sometimes-conflicting functions of social media across the epidemic-response continuum [6]. For example, the influence of social media upon attitudes, norms, and behaviours can undermine public health, with social media providing a medium for risky behaviour. Alternatively, social media can be used to react to misinformation through creation and dissemination of inoculating messages, enable real-time surveillance around disease incidents, it can promote health messages and increase access to screening or treatment. Given high levels of public social media use, intervening with health information, programmes and policies within these spaces is important to promote public health [7].

Further to the growing relationship between public health and social media, there is increasing recognition of understanding how to work with communities directly to improve health and wellbeing [8]. While the COVID-19 pandemic limited conventional community engagement because of social distancing, it also provided an opportunity to embrace alternative forms of community engagement [1,9]. Gilmore et al. [10] described digital methods for community engagement during the pandemic, including involvement of community governance systems and community health workers in garnering acceptance for quarantine measures in China [11].

Sykes et al. [12] described how citizens became not only passive consumers of information online, but actively sought information about services, symptoms, and resources, conversing about their own or others' behavioural actions. Distributed health literacy online can act as a buffer for low levels of functional health literacy [13] but can also lead to the spread of misinformation [14] and creation of ‘infodemics’ [9,10] where far-reaching spread of information can overwhelm the public [15]. The public needs information tailored to the social context in which decisions are made and protective actions taken [9].

Health agencies are becoming aware that the public increasingly see trusted individuals within their social media networks as authoritative sources of information and, when information is disseminated, it often increases its perceived legitimacy [15]. This contrasts with traditional media outlets with clearer responsibilities related to information verification and sharing. Public health agencies are also recognising the value of social media in combatting misinformation [16] and informing and mobilizing the public during health crises [[17], [18], [19]]. This type of collaboration through an online community is described as a loose knowledge collaboration [20].

This article will explore challenges and opportunities in an intervention combining expert advice from public health with community knowledge and leadership. It reports on an initiative, Essex Coronavirus Action/Support (ECAS) now known as Essex Is United, that was developed between a local authority public health team and local group administrators. During the pandemic, Essex County Council set up a Facebook group and page curated by local group administrators, with expert advice provided by the public health department. Work was completed to map all existing Facebook communities and influencers in the local area and to engage with them. The group administrators were influential online leaders by virtue of having a local and national following and high network centrality [21].

Opportunities presented by the collaboration can be understood in terms of Bourdieu's concept of social capital. Bourdieu defined social capital as “the sum of the resources, actual or virtual, that accrue to an individual or a group by virtue of possessing a durable network of more or less institutionalized relationships of mutual acquaintance and recognition” (p.119) [22,23]. He identified different forms of capital, including social, symbolic and economic capital [22,24]. The principal contribution that the local authority brings to the ECAS collaboration is its status as a recognised authority; a form of symbolic capital. Bourdieu defines symbolic capital as “the form that the various species of capital assume when they are perceived and recognised as legitimate” [24]. Social capital brought to the collaboration by local group administrators are their social networks and communities. These communities are based on shared identity and an informal logic that includes recognised individuals, local symbols, and a sense of warmth. A key strength is engagement but the challenge for group administrators is that they do not normally have access to symbolic capital to be accepted as trusted sources of information in the same way as public authorities. Thus, working together the local authority brings symbolic capital, whilst local group administrators bring social capital.

The current study examines the question: how does collaboration between public health professionals and local social network group administrators create opportunities and challenges for community engagement during a global crisis?

## 2 Methods

The study involved semi-structured interviews with three groups of stakeholders based upon a purposive sampling strategy: members of the core public health department who were involved in developing the initiative, experienced Facebook group administrators who managed, organised, and moderated the group online, and other staff within the wider public health department (N = 14). To protect participants' identities, exact numbers per group cannot be reported.

Participants were identified through the local authority and approached via email with information sheets. They were interviewed on a digital video platform by one of three experienced researchers between November 2021 and May 2022. Interviews lasted between 45 min and 2 h. The topic guide covered awareness of the initiative and any role undertaken, initiative aims, strengths and weaknesses, perceptions of the initiative, processes of creating content and community building, and contextual factors. The topic guide can be accessed at https://osf.io/va563/. Study documentation was developed through reflective discussion meetings between researchers and informed by Public Involvement and Engagement (PIE) representative feedback.

Interviews were transcribed and coded using NVivo 12 qualitative software. Data were analysed using framework analysis [25]. A coding framework was developed by the three interviewers, who then coded the data. Following familiarisation with the data to identify key ideas, the researchers developed analytical structures, largely built upon emergent concepts. The framework was applied to all data, refined, ordered, and interpreted.

This dataset contributes to a larger mixed methods evaluation, which is being published separately (see [link redacted for anonymity purposes])

## 3 Results

At the very early stages of the pandemic, members of Essex County Council drew on personal connections to form a collaboration with local group administrators to establish a Facebook group and page. During the first lockdown in England in March 2020, there was significant traffic and demand to the page, with 13,000 communications from the public in the first week [26]. The group had approximately 37,000 members at the time the study protocol was written. To address how the collaboration between public health professionals and local group administrators created both opportunities and challenges, findings are presented according to two principal themes which emerged from the analysis: ‘collaboration as opportunity’ and ‘collaboration as challenge’.

### 3.1 Theme 1: collaboration as opportunity

The local authority, as a recognised institution, has symbolic capital and values around public accountability, impartiality, and fairness. However, local authority participants highlighted the challenge of negative public perceptions of local government that are a barrier to engaging with the public:

*“People don’t love the local authority, they really don’t, they think we’re about dog poo and potholes and taking your children away”* (Core public health staff, participant 9)

A group administrator highlighted challenges for local authorities to engage through social media directly:

*“‘County council Facebook pages and Twitter accounts are just boring, they can put as many emojis and funny pictures as they like, people do not look at them for that sort of thing, they just look what day their bin’s going to be picked up and that sort of thing’”* (Group administrator, participant 12).

Involvement of the local authority was important in securing a ‘blue tick’ verification badge from Facebook (indicating a trusted site). Wider public health members indicated local authority involvement enabled added legitimacy and credibility:

*“… they had a hotline to expert views. So, Essex Coronavirus actually could get the most up-to-date evidence-based information underwritten by Essex County Council, Public Health. So, it had a degree of kudos in what it was saying”* (Wider public health staff, participant 1).

The collaboration enabled a more creative way of working that was both credible, nimble, and responsive:

*“The advantages were access to information, credibility … being able to go to the head of the NHS in Essex … It made us very nimble; it made us very fast … If we’d gone in there and we’d said, ‘Well, this is the answer to your question.’ And they come back and said, ‘Well, how do you know? Who are you? You’re just a Facebook admin.’ Correct. When I can say, ‘I got this directly from Public Health’, we’re golden”* (Group administrator, participant 13).

*“They [ECAS] could move at pace and get messages out instantly. So that sort of speed: that kind of creativity as well, that they showed, I think was very, very powerful at the start and I don’t think our more traditional routes could respond in the same way”* (Wider public health staff, participant 1).

A key consequence of the collaboration was greater freedom in the ways of communicating and engaging that were regarded as legitimate. Humour was employed to capture people's attention, imagination and promote discussion. For example, a cartoon character called ‘Barry the seagull from Southend’ was created to reinforce messages and build rapport (see blog available at [link redacted for anonymity purposes]).

Participants praised the responsiveness of administrators, speed and agility of dissemination, challenging of misinformation, and consistency in moderation. Additionally, personalities and creativity of key administrators who produced high quality content and possessed good conflict management skills were recognised. Team members showed great commitment and worked around the clock, particularly when the intervention started. Finally, participants described the local authority as being unusually open to innovative approaches in terms of willingness to hand over power and responsibility. The public health team indicated that they recognised the need for such an intervention and therefore invested time, energy and financial resources required.

### 3.2 Theme 2: collaboration as challenge

For the local authority, the concerns were that its symbolic capital was at risk by being perceived as acting outside the conventional ‘corporate’ approach. For example, the use of humour by group administrators to promote engagement posed a perceived threat to the local authority's symbolic capital by risking transgression of corporate communication rules and, at various points, local authority staff expressed concern. Participants advised that humour should be used sensibly, tactically, and selectively to avoid complaints or dissatisfaction related to content. Initial concerns regarding reputational damage were discussed within interviews, such concerns were reportedly reduced as the local authority gained more understanding of how the online community managed itself:

*“I think initially the council was worried about micromanaging what people were saying in terms of reputation, but they realised that actually if you managed it properly, obviously there would be some ‘admining’ of things that were untrue or hurtful but ultimately, communities self-manage themselves”* (Group administrator, participant 14).

For group administrators, the concern was that their social capital in the form of their reputation and credibility may be at risk if they are perceived as mouthpieces of the local authority. Indeed, participants identified that, when the initial mapping exercise was completed, approximately half of local group administrators were reluctant to engage with the local authority for this reason.

Many participants were keen to emphasize that the initiative was an amplifier and adjunct to the local authority communication team, rather than a competitor or replacement:

*“It won’t work as single means: not by a long, long way because the vast majority of people don’t look at Facebook and they don’t look at that Facebook group in Essex … it’s one aspect of a whole raft of interventions that are going on to try and inform, convince, change, cajole, nudge people into different behaviours”* (Wider public health staff, participant 1).

Participants within the wider public health team commented that the initiative might not reach those unconcerned with health risks, and may simply become, what some participants referred to as an *“echo chamber”,* of similar people voicing similar views. While those who developed the initiative generally accepted this, they also highlighted that the approach is intended to augment rather than replace other approaches. Specifically, they identified three alternative viewpoints. First, alternative networks for engaging the public (e.g., voluntary sector), faced the same challenges of communities being self-selected. Secondly, they argued that those using the group are embedded within wider family and friendship networks and could learn how to have difficult conversations with their own family and friends and address misinformation from others:

*“[Members] are knowledge nodes … they trust us so they are then going to be able to go out into their communities and talk to the people that we can’t reach, who aren’t ever going to be members of our group because they don’t trust us: So, what we have to do is empower people in the group with knowledge … teach them how to have these conversations with people who don’t agree with them”* (Group administrator, participant 13).

Thirdly, the core public health team argued that a range of social media platforms could be used. Although ECAS do manage a Twitter account, some thought if the intended audience was, for example, teenagers unconcerned with health risks, alternative platforms such as TikTok would be more appropriate. Messages in this case could be targeted at emphasizing how teenagers’ behaviour impacts on vulnerable loved ones, such as grandparents.

## 4 Discussion

This study sought to understand how public health professionals can use social media to improve engagement with the public and build online communities to inform and support during a pandemic. While the role of social media in delivering public health messages has received growing attention, how public health professionals use social media collaborations in creative and innovative ways is less developed.

The study found a collaboration between public health professionals and local group administrators presents both opportunities and challenges. The collaboration combined symbolic capital of the local authority with the social capital and networks of local administrators to engage local people in online communities. This proved effective in building trust and a sense of ownership within its members (see wider efficacy evaluation.

[link redacted for anonymity purposes]). However, the collaboration also increases perceived risk of damage to each parties' social capital, through reputational risk. Each form of capital has its own logic and is judged by different criteria so combining different forms can lead to perceived risks. Heldman et al. [2] warned “when reaching out to and/or partnering with group administrators as they often achieve such a status because they are perceived as independent and trustworthy, public health organizations must be sensitive to the possible risk of influencers becoming or being seen as ‘spokespersons’ for their organizations” (p.7).

Effective collaboration requires commitment at a senior level and strong leadership within the collaboration from people who understand both local authority organisational culture and the culture of social media networks. This ‘bicultural’ leadership involves defending the independence of the initiative while navigating political sensitivities. Importantly, the current study identified the positive role social media can play in engaging the public and building community. The results support previous studies highlighting the role of social media in combatting misinformation [16] and identifying how trusted individuals within social media networks are seen as authoritative sources of information [15].

There are limitations to the study to consider. First, the sample size is relatively modest, however, it did consist of all key stakeholders. A second potential limitation is the extent to which findings are generalisable to other locales. The local authority covers a large geographical area including rural and semi-rural areas with several large towns. Yet, participants did not identify any geographic or social factors unique to the local area that might limit the transferability of the initiative. Rather, they identified characteristics of those developing and leading the initiative who needed strong motivation, good skills in managing the tensions in the collaboration and specific skills in producing engaging content and managing conflict.

There are several implications; in practice, social media collaboration offers a different, adjunct, way of working to the traditional ‘broadcast’ model of communication focused upon delivering health messages to a mass audience. At the heart of this was building online communities using new ways of communicating that were more natural (where people already are) and interactive. Other work on ECAS reinforces this [27,28]. Participants emphasised that the intervention could address general public health issues and be integrated with traditional channels of communication. In line with this, the initiative has branched out creating new groups to address issues such as the cost-of-living crisis, climate change strategy and the Ukraine crisis. Finally, there are implications for professional training of public health professionals in terms of supporting skills needed for this type of work. As well as skills involved in using social media and communication, it also includes a wider issue about reframing the relationship between public health professionals and the public.

## 5 Conclusions

The central message of the study is that social media can extend beyond providing new channels of communication for the delivery of public health messages to enabling a new way of working that allowed for long-term engagement and online community building. The study contributes to the field through examining how a collaboration between a local authority public health team and local group administrators can contribute towards community engagement and examines the opportunities and challenges that it presents. This evaluation will inform future delivery of the digital community development approach. Whilst challenges should be considered, dynamic approaches show promise for expanding the reach of public health messages.

## Ethical approval

Ethical approval was obtained from London South Bank University Health and Social Care Ethics Panel (Reference ETH2021-0149). Participation in the study was based on the active and informed consent of all research participants. Standard ethical principles were followed regarding informed consent, anonymity, conditional confidentiality, right to withdrawal and data protection. The consolidated guidelines for reporting qualitative research were followed [29].

## Author contributions

The Public Health Intervention Responsive Studies Team (PHIRST) South Bank conceived the evaluation in collaboration with the local authority partner, all authors contributed to this process. XX and XX refined the design of the current study. XX, XX & XXX coordinated data collection, analysed and interpreted the data, and wrote the first draft of the manuscript. The submitted version has been prepared and approved by all authors.

## Funding

This work was supported by the National Institute for Health and Care Research (NIHR; PHIRST South Bank Award ID NIHR131568 and Research Award ID NIHR134151). The views expressed are those of the author(s) and not necessarily those of the NIHR or the Department of Health and Social Care.

## Data availability statement

The raw dataset will not be made publicly available as this cannot be fully anonymised.

## Declaration of competing interest

The authors declare the following financial interests/personal relationships which may be considered as potential competing interests: Authors, unless specified, do not have any commercial or financial relationships which could be construed as resulting in competing interests. DJF owns shares in equity trading funds which in turn have holdings in Meta and SS has a personal private pension with index tracker investments including in Meta.
